# Curcumin protects neural cells against ischemic injury in N2a cells and mouse brain with ischemic stroke

**DOI:** 10.1002/brb3.921

**Published:** 2018-01-22

**Authors:** Cai‐Jun Xie, Ai‐Ping Gu, Jun Cai, Yi Wu, Rui‐Cong Chen

**Affiliations:** ^1^ Department of Neurosurgery Guangdong Provincial Hospital of Chinese Medicine Guangzhou China; ^2^ Department of Ophthalmology Guangdong Second Provincial General Hospital Guangzhou China

**Keywords:** apoptosis, bax, cerebral ischemia/reperfusion, curcumin, oxygen‐glucose deprivation and reoxygenation

## Abstract

**Background and Purpose:**

Curcumin, a natural antioxidant isolated from Curcuma longa, has been reported to exert neuroprotective effect in animal models of ischemic stroke. However, the underlying mechanism is still not fully understood. The purpose of this study was to investigate the effect of curcumin treatment on neuronal apoptosis in the periinfarct cortex after cerebral ischemia/reperfusion (I/R) injury and in mouse N2a cells after oxygen‐glucose deprivation/reoxygenation (OGD/R) injury and its underlying mechanism.

**Methods:**

The cerebral I/R injury was established by 1‐hr middle cerebral artery occlusion (MCAO) and reperfusion in mice. Infarct volume was determined by TTC staining, and neurological score was evaluated by mNSS. Cell morphology in the ischemic boundary zone were detected by HE staining. The number and apoptotic rate of neurons in ischemic boundary zone were assayed by immunohistochemistry and TUNEL, respectively. Mouse neuroblastoma N2a cells were subjected to OGD/R. Cell viability was assessed with CCK‐8. The mitochondrial membrane potential was measured using JC‐1 staining. The expression of Bax, Bcl‐2, and caspase‐3 was detected using Western blotting. Besides, cellular distribution of Bax was determined by immunofluorescence assays.

**Results:**

Curcumin treatment reduced infarct volume, improved neurological function, alleviated the morphological damage of neurons, and increased neuronal survival rate after I/R injury *in vivo*. Moreover, curcumin treatment improved cell viability, reduced cell apoptosis, increased Bcl‐2 protein levels while decreased Bax and caspase‐3 expressions in mouse N2a cells after OGD/R injury. Besides, curcumin treatment inhibited Bax activation and maintained mitochondrial membrane integrity.

**Conclusion:**

Curcumin promotes neuron survival *in vivo* and *in vitro* to exert neuroprotective effects against ischemia injury. Moreover, our results for the first time demonstrated curcumin inhibited ischemia‐induced mitochondrial apoptosis via restricting Bax activation, which may be one of the possible mechanisms underlying the neuroprotective effects of curcumin.

## 1 INTRODUCTION

Stroke is a leading cause of death and permanent adult disability all over the world and remains a major challenge to public health (Correction to Heart Disease and Stroke Statistics‐2017 Update: A Report From the American Heart Association,” 2017). One subtype of stroke, cerebral ischemia, is a complex pathophysiologic process, during which excitotoxicity, oxidative stress, inflammation, and dysfunction of ATP production are involved (Benakis, Garcia‐Bonilla, Iadecola, & Anrather, 2014; Connolly, Dussmann, Anilkumar, Huber, & Prehn, 2014; Jung et al., 2010; Naito & Yoshikawa, 2006). These pathophysiologic events overlap and intercommunicate, eventually resulting in neuronal death.

Apoptosis is one of the major pathways that leads to neuronal death after cerebral ischemia/reperfusion (I/R), and mitochondria are the cellular organelles that play important roles in the apoptotic signaling in ischemic injury (Mattson, Duan, Pedersen, & Culmsee, 2001; Zuo et al., 2014). In response to cerebral I/R, mitochondria release free radicals and overproduce reactive oxygen species, which in turn impair mitochondrial function and result in loss of mitochondrial membrane potential (Kowaltowski, Castilho, & Vercesi, 2001; Nita et al., 2001). In addition, during the process of cerebral ischemia, a proapoptotic protein, Bax, is activated and then translocated to mitochondria, leading to mitochondrial membrane permeabilization (Kroemer, 2003). Subsequently, the permeabilized mitochondria release cytochrome *c*, which activates caspases and results in cell death after I/R (Kuwana et al., 2002). While in healthy cells, Bax is primarily in the cytoplasm and controlled by the antiapoptotic protein Bcl‐2 (Cao et al., 2001).

Curcumin, mainly extracted from the root of the Curcuma longa Linn, has been reported to confer a range of pharmacological effects, including anticancer, anti‐inflammation, antioxidation and antivirus effects (Guo et al., 2013; Kunwar, Barik, Sandur, & Indira Priyadarsini, 2011; Manca et al., 2015; Mazzarino et al., 2015). Curcumin also exerts neuroprotective effects against Alzheimer's disease, Parkinson's disease, depression, and cerebral ischemia (Liu et al., 2014; Lopresti et al., 2015; Miao et al., 2016; Rathore et al., 2008; Wang et al., 2005; Wu, Ying, & Gomez‐Pinilla, 2006; Zhang et al., 2017; Zhao et al., 2008, 2010). However, the exact mechanisms underlying the protective effects of curcumin against ischemic stroke are still not fully understood. Therefore, this study was designed to investigate the mechanism underlying curcumin protecting against neuronal cell death in the OGD/R‐treated mouse neuroblastoma (N2a) cells and in the cerebral I/R mice.

## 2 MATERIALS AND METHODS

### 2.1 Animals and MCAO surgery

Male C57BL/6 mice (7–8‐week‐old), weighing 20–25 g, were obtained from Medical Laboratory Animal Center of Guangdong (Guangzhou, China). All animal experiments and procedures were approved by the Institutional Animal Care and Use Committee of the Guangdong Provincial Hospital of Chinese Medicine (Guangzhou, China). The mice, which were housed in the animal room at 22–24°C with 12‐hr light/dark circle and free access to food and water, were randomized into sham group (SHA, *n *= 8), MCAO treated with vehicle group (VEH, *n *= 8) and MCAO treated with curcumin group (CUR, *n *= 32).

For the MCAO surgery (Pan et al., 2017), mice were anesthetized with 4% isoflurane (Ruiwode, Shenzhen, China). Then, the focal cerebral ischemia was produced by intraluminal occlusion of the right middle cerebral artery using a silicone coated nylon (6.0) monofilament (Doccol Corporation, Redlands, CA, USA). One hour later, the occluding filament was withdrawn to allow blood reperfusion. Cerebral blood flow was monitored with laser Doppler flowmetry (PeriFlux 5000; Perimed AB, Sweden). A drop in regional cerebral blood flow (CBF) below 30% from baseline after the insertion of the filament was considered to be sufficient for induction of focal cerebral ischemia. The core body temperature of mice was maintained at 37.0 ± 0.5°C using a thermostatically controlled heating blanket connected to a thermometer probe in the rectum, and at the same time, the physiological parameters including heart rate (HR), breathing rate (BR), and temperature were monitored during surgery. Mice in the SHA group were treated identically, except that the middle cerebral arteries were not occluded.

### 2.2 Drug administration

In dose‐response study, mice of CUR group were intraperitoneally injected with curcumin (Sigma‐Aldrich, USA) dissolved in normal sterile saline with 1% dimethyl sulfoxide (DMSO) at a dose of 100, 200, 300, 400 mg/kg, immediately after 1 hr of occlusion. Both VEH and SHA groups were injected with the same volume of sterile saline with 1% DMSO.

### 2.3 Cell culture and OGD/R injury

Mouse N2a cells were cultured in Dulbecco's modified Eagle's medium (DMEM) containing 10% fetal bovine serum and 4.5 g/ml glucose with 100 μg/ml penicillin and 100 μg/ml streptomycin in normoxic conditions (5% CO_2_ and 21% O_2_) at 37°C. To initiate OGD, N2a cells were incubated in serum and glucose‐free DMEM and saturated with 5% CO_2_ and 95% N_2_ at 37°C for 3 hr. After OGD exposure, the cells were subjected to reoxygenation with DMEM containing serum and glucose in normoxic conditions for 24 hr. Cells were treated with 5, 15, 25, and 35 μmol/L curcumin or DMSO (as control) for 24 hr while being reoxygenated. Control cells were cultured in DMEM in normoxic conditions.

### 2.4 Neurobehavioral assessment

Behavioral tests were performed to assess the neurological deficits, based on the modified neurological severity scores (mNSS) at 24 hr after MCAO by two investigators who were blinded to the experimental groups, as described previously (Pan et al., 2017). Neurological function was graded on a scale of 0 to 18 (normal score, 0; maximal deficit score, 18). In the severity scores of injury, 1 score point is awarded for the inability to perform the test or for the lack of a tested reflex; thus, the higher score, the more severe is the injury.

### 2.5 Measurement of infarct size

At 24 hr after MCAO, mice were deeply anesthetized with 10% chloral hydrate and then decapitated. Brains were rapidly removed, and 1‐mm thick coronal sections from throughout the brain were stained with 2% 2,3,5‐triphenyltetrazolium chloride (TTC; Amresco Inc., Solon, OH, USA) to evaluate the infarct volume, as described previously (Pan et al., 2017). Infarct volume was manually quantified using Image J (Bethesda, MD, USA) in a blinded way and expressed as a percentage of the contralateral structure.

### 2.6 Hematoxylin and eosin (HE) staining

Cell morphology in the ischemic boundary zone of brain (Figure 3a) were measured by HE staining using Hematoxylin and Eosin Staining Kit (Beyotime, China), according to the manufacturer's instructions.

### 2.7 Immunohistochemistry

Coronal brain sections (5‐μm thick) were randomly selected between 0 and 1 mm posterior to the bregma. After deparaffinization and rehydration, the sections were incubated for 15 min at 95°C in 0.01 mol/L citrate buffer (pH 6.0) for antigen retrieval. Following incubation in 0.3% H_2_O_2_ followed by 5% bovine serum albumin to avoid nonspecific immunoreactions, the sections were stained overnight at 4°C using anti‐NeuN antibody (ab104224; Abcam). Then, the sections were washed with 0.01 mol/L PBS and later incubated using a corresponding secondary antibody (8125; Cell Signaling Technology) for 1 hr at room temperature; the immunoreactivity was visualized by treatment with Dako Envision kit HRP (K4006; DAKO). Finally, counterstaining was carried out with hematoxylin, dehydrated, mounted. NeuN‐positive cells were observed using 3,3′‐diaminobenzidine‐enhanced liquid substrate system and quantified using a bright microscope (Olympus, CX21FS1, Japan).

### 2.8 Western blotting

Cellular samples were lysed with NP40 lysis buffer (P0013F; Beyotime, China) and then subjected to SDS‐PAGE and immunoblotting as previously described (Cai et al., 2014). Primary antibodies against Bax (M1312; HuaAn Biotechnology, China), Bcl‐2 (ab692, Abcam), cleaved caspases‐3 (ab2302; Abcam), GAPDH (ab8245; Abcam) were used.

### 2.9 Mitochondria membrane potential measurement

Cells were stained with JC‐1 dye (Beyotime, China) to detect a change in mitochondrial membrane potential, according to the manufacturer's instructions. Due to that, mitochondrial membrane potential is high in normal cells; JC‐1 accumulates in the mitochondrial matrix to form red fluorescent JC‐1 aggregates. When cells undergo apoptosis, the mitochondrial membrane potential is low which prevents JC‐1 accumulation in the mitochondria, and thus, the dye is dispersed throughout the entire cell leading to a shift from red (JC‐1 aggregates) to green fluorescence (JC‐1 monomers) (Wang, Ownby, Zhang, Yuan, & Li, 2010). Briefly, cells were cultured in 12‐well plates. After OGD/R injury or for 24 hr, cells were stained with JC‐1 (2 mmol/L) and incubated in the incubator (37°C, 5% CO_2_) for 20 min. Following incubation, cells were washed once with PBS and then the fluorescence was detected with a fluorescence microscope. JC‐1 aggregates and monomers were detected with an excitation wavelength of 525 nm or 490 nm and emission wavelength of 590 nm or 530 nm, respectively. Fluorescent intensity was examined with a fluorescence spectrophotometer. The ratio of JC‐1 aggregates to monomer was calculated as an indicated of mitochondria membrane potential.

### 2.10 Cell viability assay

N2a cells cultured in 96‐well plates were exposed to 3‐hr OGD and 24‐hr reoxygenation. Cell viability was measured using the Cell Counting Kit‐8 (Beyotime, China), according to the manufacturer's instructions.

### 2.11 TUNEL assay

TUNEL technique was used to detect cell apoptosis via an in situ cell death detection kit (Roche, Germany) according to the protocol of the kit. An immunofluorescence analysis of neurons with antibody against the neuronal marker protein‐NeuN, TUNEL staining, and DAPI (Beyotime, China) staining was performed in the ischemic boundary zone of brain (Figure  3a) or OGD/R‐injured N2a cells to calculate the apoptotic cells. Three nonoverlapping visual fields were chosen randomly within the regions of interest. A minimum of 300 cells were counted, and those cells with NeuN, TUNEL‐positive, and intense chromatin clumping (DAPI staining) were counted as neuronal apoptotic cells. Results were expressed as percentage of TUNEL‐positive cells relative to DAPI‐positive cells.

### 2.12 Immunofluorescent

N2a cells were grown overnight into 35‐mm glass bottom dishes and then treated with DMSO or curcumin (25 μmol/L) for 24 hr after OGD/R. Cells were fixed with 4% paraformaldehyde, permeabilized with 0.5% Triton X‐100 in PBS, and incubated at 4◦C with anti‐Bax antibody overnight. Cells were then incubated with FITC‐conjugated‐secondary antibodies (KPL) at 37°C for 1 hr. Cellular nuclei were stained with DAPI for 5 min and viewed by an LSM780 laser scanning confocal microscopy (Carl Zeiss).

Brains from experimental rats were fixed by transcardial perfusion with saline, followed by perfusion and immersion in 4% paraformaldehyde. Sections were deparaffinized in xylene in two steps, each lasting 15 min; then dehydrated with 100% ethanol in two steps lasting 3 min each; and finally hydrated with 95% ethanol for 1 min. Sections were incubated in 1 mol/L HCl for 30 min at 37°C, blocked for 30 min at 4°C with PBS containing 5% donkey serum and 0.25% Triton‐X100, and then incubated for 16 hr with the following primary antibodies: anti‐NeuN antibody, and anti‐Bax antibody. Next, sections were incubated for 2 hr at room temperature in the dark with the mixture of FITC‐conjugated secondary antibodies and Alexa Fluor^®^ 546‐conjugated secondary antibodies (Invitrogen) at 37°C for 60 min. Sections were washed in the dark three times with PBS for 10 min each time. Sections were sealed under coverslips using Antifade Solution (Vector Laboratories, USA) and analyzed using a fluorescence microscope.

### 2.13 Statistical analysis

The data are shown as the means ± the standard error of the means (SEMs). A one‐way analyses of variance (ANOVA) followed by a Student, Newman‐Keuls, or Dunnett's *posthoc* test were utilized for the comparisons between more than two groups. Besides, mortality rate was compared by chi‐square test. SPSS 18.0 (SPSS, Chicago, IL, USA) was used for the statistical analyses, and the statistical significance was set at *p < .05 and **p < .01.

## 3 RESULTS

### 3.1 Laser Doppler flowmetry monitor confirms the uniformity of MCAO models

**Figure 1:**
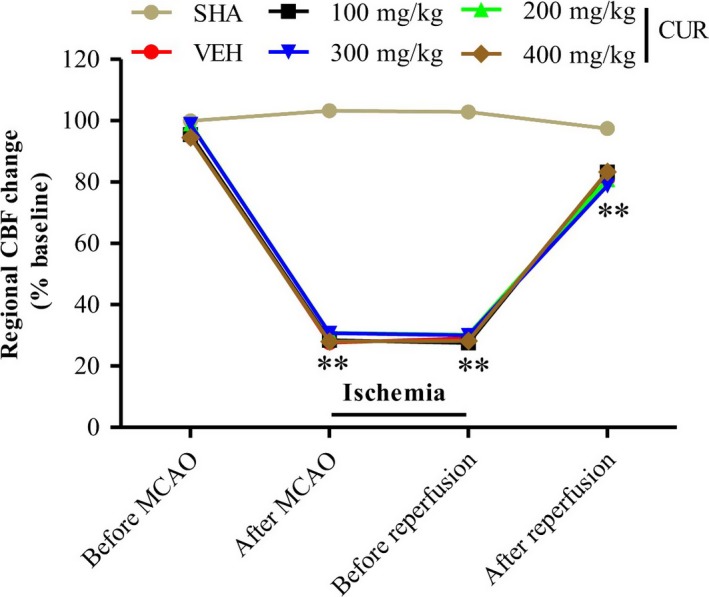
Laser Doppler flowmetry monitor confirms the uniformity of cerebral I/R models. The changes of CBF during cerebral I/R were monitored with laser Doppler flowmetry. CBF was normalized by comparison to the mean CBF before administration of MCAO in mice. There was no difference in the decrease in CBF during MCAO between animals assigned to different treatment groups. Data are means ±SEM (*n *= 8 per group); *p < .05 vs. SHA group, **p < .01 vs. SHA group

All animals of this study showed similar values for BR, HR, and temperature (Table 1). The laser Doppler flowmetry signal showed that regional CBF was reduced equivalently in VEH and CUR groups, but not in SHA group during ischemia (Figure 1), confirming the uniformity of MCAO models.

**Table 1:** Physiological parameters

	SHA	VEH	CUR			
			100 mg/kg	200 mg/kg	300 mg/kg	400 mg/kg
Before Ischemia						
HR	400.2 ± 22.8	403.6 ± 14.9	412.5 ± 23.6	399.1 ± 23.8	402.4 ± 17.7	408.3 ± 12.8
BR	78.7 ± 7.6	73.2 ± 8.7	80.2 ± 4.7	71.3 ± 6.7	75.3 ± 5.1	73.9 ± 7.2
T	37.1 ± 0.4	37 ± 0.3	37 ± 0.2	37.1 ± 0.3	37.1 ± 0.1	36.9 ± 0.2
After Ischemia						
HR	402.8 ± 19.6	407.4 ± 17.6	410.4 ± 16.9	413.0 ± 11.3	401.4 ± 22.7	401.3 ± 21.6
BR	71.6 ± 11.2	74 ± 8.7	73.6 ± 9.3	79.8 ± 3.2	76.2 ± 4.7	70.2 ± 12.3
T	37 ± 0.2	37.2 ± 0.1	37 ± 0.1	37.2 ± 0.3	37.1 ± 0.2	37.0 ± 0.2
Before reperfusion						
HR	427.1 ± 21.8	432.2 ± 27.4	441.9 ± 20.1	437.3 ± 17.9	422.9 ± 30.2	429.8 ± 27.6
BR	102.3 ± 7.8	104.2 ± 6.9	108.7 ± 5.1	111 ± 7.7	100.1 ± 10.2	99.5 ± 14.2
T	37.2 ± 0.3	39.2 ± 0.2	38.9 ± 0.3	38.5 ± 0.5	38.7 ± 0.4	39 ± 0.3
After reperfusion						
HR	431.2 ± 26.8	430.4 ± 23.3	434.5 ± 19.9	427.9 ± 31.3	441.3 ± 16.5	428.9 ± 24.6
BR	97.3 ± 15.2	101.4 ± 9.7	107.3 ± 8.6	105.8 ± 10.5	102.8 ± 12.2	107.2 ± 11.1
T	37 ± 0.3	39 ± 0.1	38.7 ± 0.4	38.4 ± 0.3	38 ± 0.5	38.3 ± 0.2

HR, heart rate; BR, breathing rate; T, temperature.

### 3.2 Curcumin attenuates I/R‐induced brain injury

**Figure 2:**
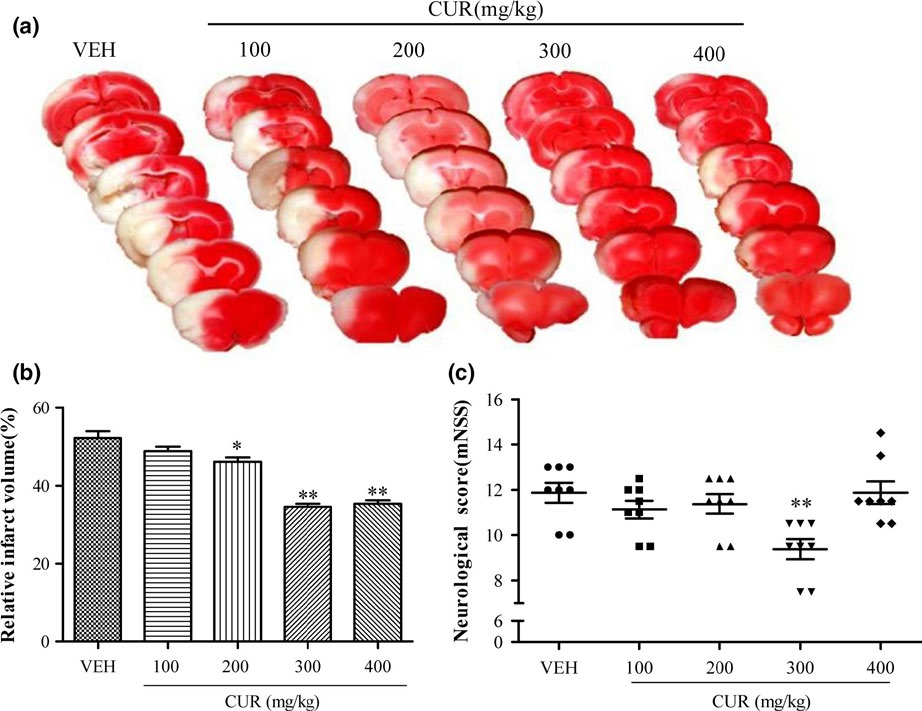
Curcumin attenuates I/R‐induced brain injury. (a) Representative TTC‐stained coronal sections of mouse brain from VEH and CUR (100, 200, 300 and 400 mg/kg) groups at 24 hr after I/R. Pale areas represent infarction. (b) Quantification of infarct volume at 24 hr after I/R. (c) Neurological scores were assessed at 24 hr after I/R. Data are means ± SEM (*n *= 8 per group); *p < .05 vs. VEH group, **p < .01 vs. VEH group

First, we examined the protective effects of curcumin against ischemic stroke by evaluating infarct volume and neurological score. The results in Figure 2a,b showed that curcumin treatment with 200, 300, and 400 mg/kg evidently reduced the infarct volume compared to the VEH group, and 300 mg/kg curcumin has the most significant effect. Furthermore, a significant improvement in neurological score was observed in ischemic mouse treated with 300 mg/kg curcumin (Figure 2c). Taken together, these results demonstrated that curcumin attenuates I/R‐induced brain injury.

### 3.3 Curcumin reduces I/R‐induced neuronal apoptosis

**Figure 3:**
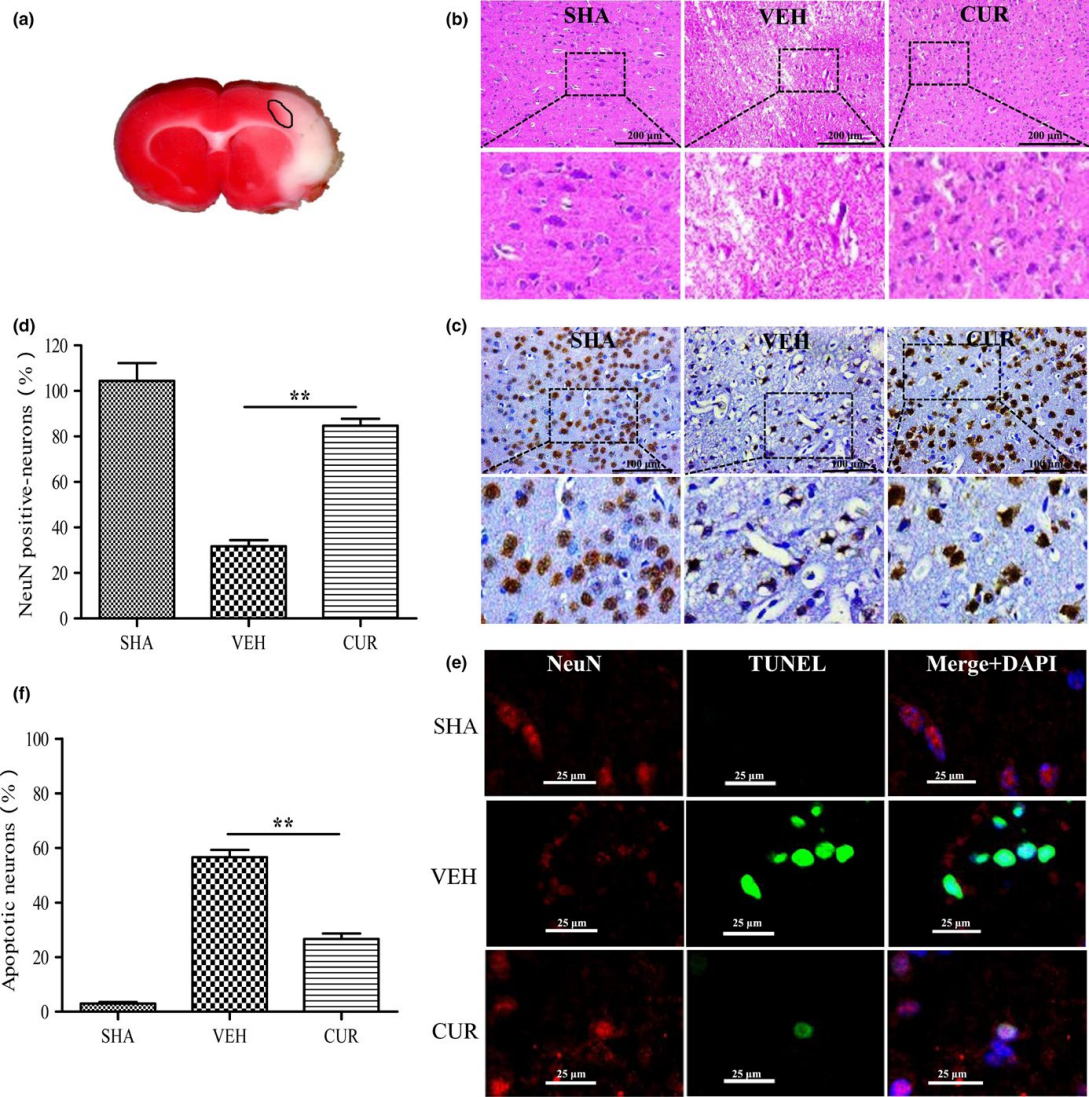
Curcumin reduces I/R‐induced neuronal apoptosis. (a) Coronal slices stained by TTC; the circle represents the periinfarct area. (b) HE staining of coronal sections in the periinfarct area (indicated by the circle (a)) of SHA, VEH, and CUR (300 mg/kg) groups at 24 hr after I/R. (c) Immunohistochemical staining for NeuN in the periinfarct area (indicated by the circle (a)) of SHA, VEH, and CUR (300 mg/kg) groups at 24 hr after I/R. (d) Quantification of NeuN‐positive cells displayed in (c). (e) TUNEL staining (green) for neuronal (red) apoptosis in the periinfarct area (indicated by the circle (a)) of SHA, VEH, and CUR (300 mg/kg) groups at 24 hr after I/R. Nuclei were stained with DAPI (blue). (f) Quantification of NeuN‐ and TUNEL‐positive cells displayed in (e). Data are means ± SEM (*n *= 3 per group); *p < .05 vs. VEH group, **p < .01 vs. VEH group

Cell morphology in the ischemic boundary zone (Figure 3a) were detected by HE staining. The result in Figure 3b shows that the cell morphology of most neurons in the VEH group displayed shrinkage, nuclear pyknosis, and vacuolization, while the neurons in the CUR group were relatively normal and showed less damage, compared to those in the VEH group. Furthermore, the number and apoptotic rates of neurons in the ischemic boundary zone were evaluated by immunohistochemistry and TUNEL assays (Figure 3c,d,e,f). In the VEH group, the number of neurons was significantly decreased and the apoptotic rate was notably increased, compared to those in the SHA group. However, curcumin treatment could significantly mitigate the reduction in neurons and inhibits neuronal apoptosis.

**Figure 4:**
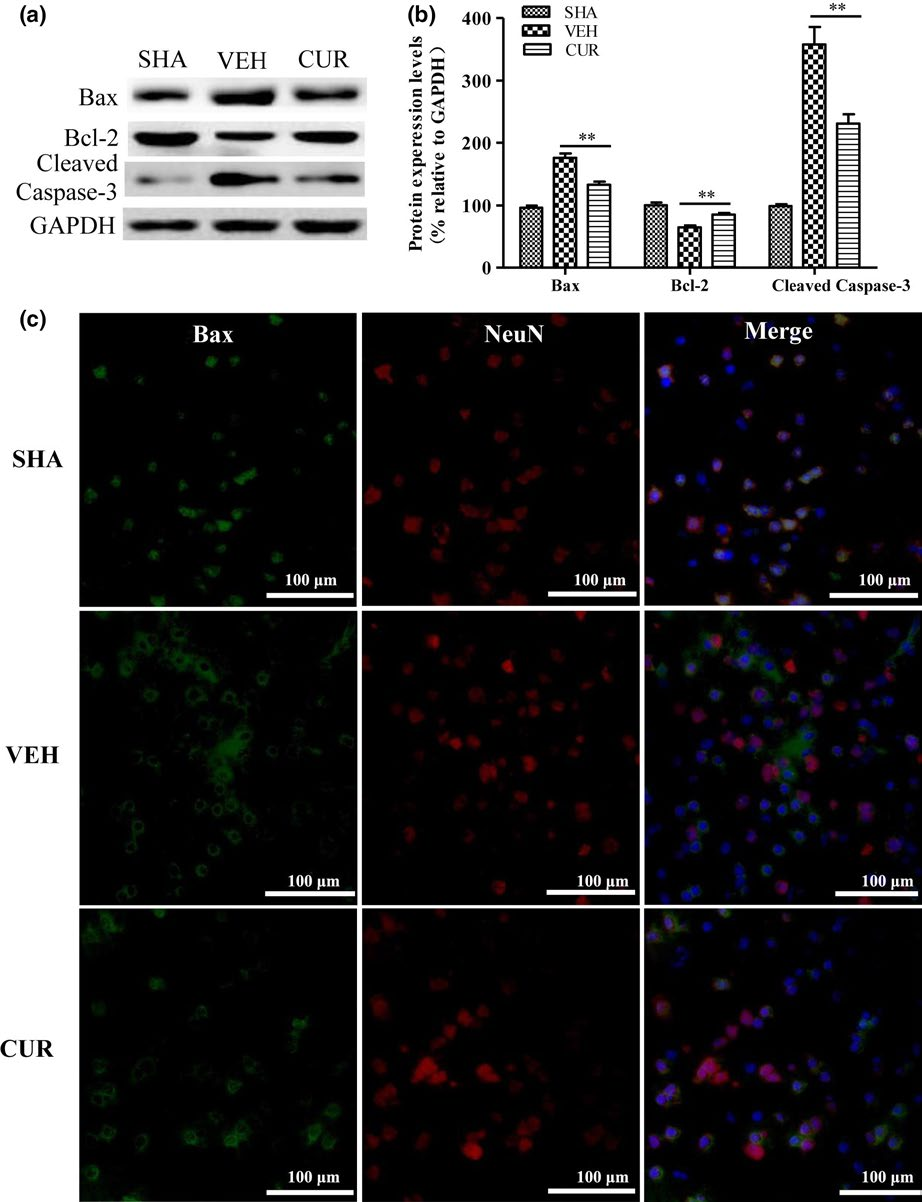
Curcumin alleviates I/R‐induced mitochondrial apoptosis pathway. (a) The protein expression levels of Bax, Bcl‐2, cleaved caspase‐3, and GAPDH in the ischemic cortex of SHA, VEH, and CUR (300 mg/kg) groups were detected by Western blotting at 24 hr after I/R. (b) Relative expression levels of Bax, Bcl‐2, cleaved caspase‐3 were calculated by normalizing to that of GAPDH, respectively. (c) Subcellular distribution of Bax (green) in neurons (red) in the periinfarct area was detected by immunofluorescent assay at 24 hr after I/R. Nuclei were stained with DAPI (blue). Data are means ± SEM (*n *= 3 per group); *p < .05 vs. VEH group, **p < .01 vs. VEH group

Furthermore, we examined the expression levels of proapoptotic protein Bax and cleaved caspase‐3, and antiapoptotic protein Bcl‐2 in the ischemic cortex 24 hr after I/R by Western blotting (Figure 4a,b). In the VEH group, the expression levels of Bax and cleaved caspase‐3 were significantly increased while Bcl‐2 expression was decreased, compared to those in the SHA group. However, curcumin treatment significantly inhibited the increase of Bax and cleaved caspase‐3 expressions, and the decrease in Bcl‐2 expression.

As the proapoptotic protein Bax plays a critical role in triggering mitochondrial apoptotic pathway (Kroemer, 2003), we further explored whether curcumin inhibits Bax activation by immunofluorescent assay. As shown in Figure 4c, Bax mainly distributed in the cell nucleus of neurons in SHM group, while after I/R, the majority of Bax translocated from the nucleus into the cytoplasm displayed in the pattern of “macaroni‐like” punctuations which are representative of mitochondrial (Perfettini, Roumier, & Kroemer, 2005; Walensky & Gavathiotis, 2011). Curcumin treatment could partially block I/R‐induced the translocation of Bax, a substantial portion of which was still distributed in the nucleus. These data showed that curcumin has effectively protective effects against neuronal apoptosis and Bax activation in ischemic brain.

### 3.4 Curcumin reduces OGD/R‐induced cell apoptosis in mouse N2a cells

**Figure 5:**
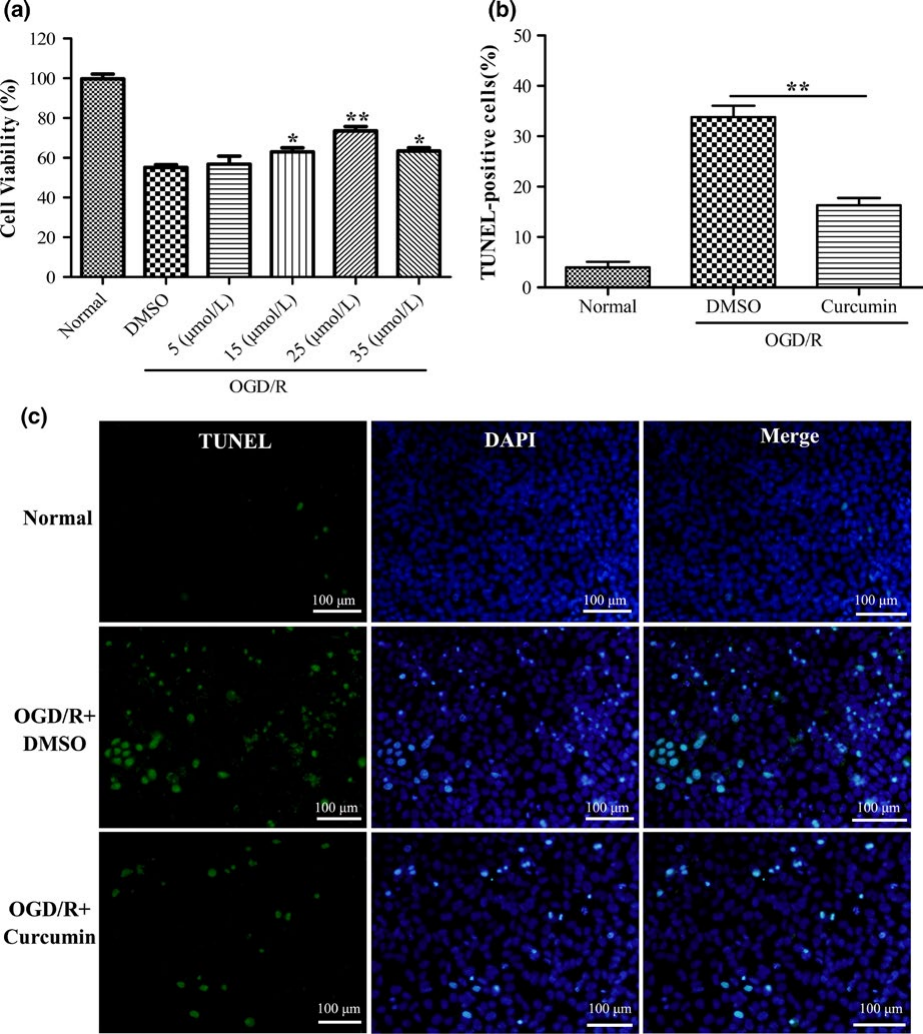
Curcumin reduces OGD/R‐induced cell apoptosis in mouse N2a cells. (a) N2a cells were treated with DMSO or curcumin (5, 15, 25, and 35 μmol/L) for 24 hr after OGD/G. Cell viability of N2a cells was detected by CCK‐8. (b) TUNEL staining (green) for N2a cell apoptosis in normal, DMSO + OGD/R, and curcumin (25 μmol/L)  + OGD/R groups. Nuclei were stained with DAPI (blue). (c) Quantification of TUNEL‐positive cells displayed in (b). Data are means ± SEM (*n *= 3 per group); *p < .05 vs. DMSO + OGD/R group, **p < .01 vs. DMSO + OGD/R group

We further investigated the protective effects of curcumin against OGD/R‐induced N2a cell death by cell viability assay (Figure 5a). The results showed that curcumin treatment with 15, 25, or 35 μmol/L, but not 5 μmol/L, effectively elevated the cell viability of OGD/R‐injured N2a cells, compared to DMSO treatment. 25 μmol/L curcumin treatment has the most significant effect (Figure 5a). Furthermore, to explore whether curcumin could affect N2a cell apoptosis, TUNEL assay was performed. As shown in Figure 5b,c, curcumin treatment with 25 μmol/L could significantly inhibit OGD/R‐induced N2a cell apoptosis, compared to OGD/R‐treated control (DMSO treatment). These data showed that curcumin has effectively protective effects against OGD/R‐induced neuronal apoptosis *in vitro*.

### 3.5 Curcumin alleviates OGD/R‐induced mitochondrial dysfunction in mouse N2a cells

**Figure 6:**
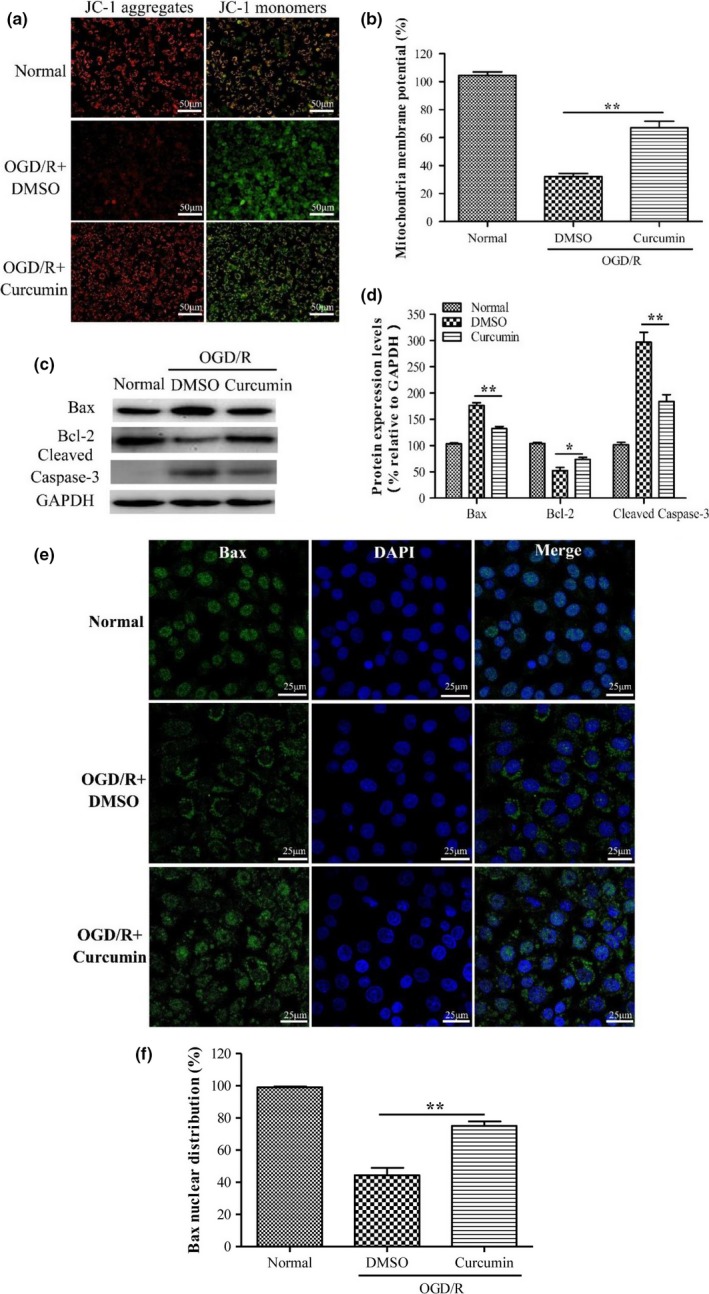
Curcumin alleviates OGD/R‐induced mitochondrial dysfunction in mouse N2a cells. (a) N2a cells were treated with DMSO or curcumin (25 μmol/L) for 24 hr after OGD/R. Mitochondrial membrane potential of N2a cells was detected by JC‐1 dying. (b) Quantification of mitochondrial membrane potential of N2a cells displayed in (a). (c) Expressions of Bax, Bcl‐2, cleaved caspase‐3, and GAPDH were detected by Western blotting. (d) Relative expression levels of Bax, Bcl‐2, cleaved caspase‐3 were calculated by normalizing to that of GAPDH, respectively. (e) Subcellular distribution of Bax (green) was detected by immunofluorescent assay at 24 h after OGD/R. Nuclei were stained with DAPI (blue). Data are means ± SEM (*n *= 3 per group); *p < .05 vs. DMSO + OGD/R group, **p < .01 vs. DMSO + OGD/R group

Mitochondria have been reported to play important roles in the apoptotic signaling in ischemic injury (Mattson et al., 2001; Zuo et al., 2014). To investigate whether curcumin has any potential protective effects on mitochondria in OGD/R‐induced N2a cell apoptosis, mitochondrial membrane potential was examined using JC‐1 dying. As shown in Figure 6a,b, OGD/R caused a significant decrease in mitochondrial membrane potential of N2a cells. However, curcumin treatment notably alleviated the decrease in mitochondrial membrane potential induced by OGD/R injury, compared to DMSO treatment. Furthermore, the expression levels of Bax and cleaved caspase‐3 were significantly increased while Bcl‐2 expression was decreased in N2a cells after OGD/R, as expected (Figure 6c,d). Curcumin treatment significantly inhibited the increase in Bax and cleaved caspase‐3 expressions, and the decrease in Bcl‐2 expression, compared to DMSO treatment.

As the proapoptotic protein Bax plays a critical role in triggering mitochondrial apoptotic pathway (Kroemer, 2003), we further explored whether curcumin inhibited Bax activation *in vitro* by immunofluorescent assay. As shown in Figure 6e,f, the majority of Bax translocated from nucleus to mitochondrial after OGD/R. Curcumin treatment could partially block OGD/R‐induced the translocation of Bax, a substantial portion of which was still distributed in the nucleus. These results showed that curcumin inhibits Bax activation and alleviates OGD/R‐induced mitochondrial dysfunction in mouse N2a cells.

## 4 DISCUSSION

The present study shows that curcumin protects neurons against apoptosis induced by cerebral I/R injury in mice and OGD/R injury in mouse N2a cells. Furthermore, curcumin alleviates OGD/R‐induced mitochondrial dysfunction in mouse N2a cells through maintaining the mitochondrial membrane potential and suppressing the upregulation expression of Bax and downregulation of Bcl‐2. In addition, our results for the first time demonstrate that curcumin inhibits Bax activation after cerebral I/R injury in the periinfarct cortex of mice and after OGD/R injury in mouse N2a cells.

Owing to the high mortality and severe neurological disorder of ischemic stroke, it is quietly urgent to find effective therapeutic drugs against ischemic injury. Curcumin, a yellow‐colored phenolic pigment obtained from the root of the Curcuma longa Linn, has been demonstrated to exert anticancer, ant‐inflammation, antioxidation and antivirus, and low toxic and side effect (Guo et al., 2013; Kunwar et al., 2011; Manca et al., 2015; Mazzarino et al., 2015), suggesting that it has good potential value in clinic. In recent years, curcumin has been used in the treatment of cerebrovascular disease due to its good antioxidation and anti‐inflammatory effects (Motterlini, Foresti, Bassi, & Green, 2000; Priyadarsini, 1997). Moreover, curcumin has been demonstrated to pass through the blood‐brain barrier of aged rats and is recommended for the prevention and treatment of Alzheimer's disease (Wang et al., 2005; Yang et al., 2005). Nowadays, a number of studies reported that curcumin improves outcomes and attenuates focal cerebral ischemic injury in MCAO rat models (Liu et al., 2016; Miao et al., 2016; Zhao et al., 2010). Similarly, our results showed that curcumin decreased the infarct volume and improved the neurological outcomes in another different MCAO mouse model, which is more and more widely used to investigate the complicated molecular mechanisms of cerebral ischemia in recent years (Evans et al., 2017; H. Liu, Zuo, & Wu, 2017; Xia et al., 2017).

While most of the studies which tried to elucidate the possible mechanisms underlying the neuroprotective effect of curcumin have focused on neuroinflammation (Bassani et al., 2017; Ullah et al., 2017), or neurotoxicity (Motaghinejad, Motevalian, Fatima, Faraji, & Mozaffari, 2017; Ramkumar et al., 2017), the importance of neuron survival has been neglected. Neurons are the core components of brain, and the morphology and function of neurons are essential to the central nervous system (Fiocchetti, De Marinis, Ascenzi, & Marino, 2013). Numerous studies reported that neuronal cell death is a critical part of cerebral ischemic stroke pathophysiology (Baczynska, Michalowska, & Witkiewicz, 2013; Rakers, Schmid, & Petzold, 2017); thus, reduction in neuron loss and improvement of neuron survival are critical for the treatment of cerebral ischemia. In the present study, on one hand, our data show that curcumin alleviates morphological damage of neurons and decreases neuron loss and apoptosis in the periinfacrt cortex after I/R injury *in vivo*; on the other hand, curcumin improves cell viability and promotes mouse N2a cell survival after OGD/R injury *in vitro*. Consistent with the previous studies on the effects of curcumin against stroke *in vivo* model (Miao et al., 2016; Zhao et al., 2010), our results further confirmed the neuroprotective effect of curcumin on stroke both *in vivo* and *in vitro* and offered a new insight into the underlying mechanism of curcumin's neuroprotective effect via promoting neuron survival.

Mitochondria are multifunctional organelles which not only play essential roles in energy metabolism and cell differentiation but also serve as an important control point in the regulation of apoptosis (Mattson et al., 2001; Zuo et al., 2014). Cerebral ischemia damages mitochondrial membrane integrity, leading to release of proapoptotic proteins into the cytoplasm, such as cytochrome *c* or apoptosis‐inducing factor, and eventually resulting in activation of cleaved caspase‐3 and apoptosis (Kroemer, 2003). Bcl‐2 family proteins are pivotal moderators of mitochondrial apoptosis through regulating mitochondrial membrane permeability (Kuwana et al., 2002; Youle & Strasser, 2008). In normal neurons, Bax is sequestered by Bcl‐2, so as to prevent the pore formation on mitochondrial membrane and inhibit apoptosis (Youle & Strasser, 2008). Upon apoptotic stimulation, Bax is activated and then translocated to mitochondria, where Bax forms oligomers resulting in loss of mitochondrial membrane potential (del Mar Martinez‐Senac, Corbalan‐Garcia, & Gomez‐Fernandez, 2001). Previous studies reported that knock‐down or inhibition of caspase‐3 provided neuroprotection via mediating neuronal death and rendering neuronal resistant to ischemic injury (Le et al., 2002). Bcl‐2 overexpression of transgenic mice significantly reduced the volume of cerebral infarction after focal ischemia, whereas Bcl‐2 knockout mice showed the increase in infarct volume (Hata, Gillardon, Michaelidis, & Hossmann, 1999; Martinou et al., 1994). These results suggest the regulation of mitochondrial membrane integrity and the importance of the Bcl‐2 protein family in cerebral ischemic injury. Our study demonstrated that curcumin upregulated the expression of antiapoptotic protein Bcl‐2 and downregulated the expression of Bax both *in vivo*, which were consistent with the results of previous study (Miao et al., 2016)*, and in vitro*. Furthermore, our results for the first time demonstrated that curcumin blocked Bax translocation to mitochondria and thus maintained mitochondrial membrane potential and prevented the activation of caspase‐3. Although the mechanisms of curcumin against ischemia injury need to be further explored, the current study suggests that promotion of neuron survival and inhibition of mitochondrial apoptotic signaling by suppressing Bax translocation may play important roles underlying this.

In summary, our study demonstrates that curcumin promotes neuron survival *in vivo* and *in vitro* to exact neuroprotective effects against ischemia injury. Moreover, our results for the first time demonstrate curcumin inhibits ischemia‐induced mitochondrial apoptosis via restricting Bax activation.

## CONFLICT OF INTEREST

The authors have no actual and potential competing interests to declare.

## AUTHORS’ CONTRIBUTIONS

All authors had full access to all the data in the study and take responsibility for the integrity of the data and the accuracy of the data analysis. Conceived and designed the experiments: RCC, CJX. Performed the experiments: CJX, APG, JC. Analyzed the data: CJX, YW, JC. Contributed reagents/materials/analysis tools: RCC, JC. Wrote the paper: JC. Revised the manuscript: RCC, APG.

## SIGNIFICANCE STATEMENT

According to the results of present study, we demonstrated the neuroprotective effects of curcumin by promoting neuron survival *in vivo* and *in vitro*. Curcumin inhibited ischemia‐induced mitochondrial apoptosis via restricting Bax activation to alleviate cerebral ischemic injury. We believe the findings will provide positive information to basic science and pharmaceutical development of cerebral ischemia stroke. We presume the findings of this study will be of special interest to the readers with background of neuroscience, clinical neurology, and neurovascular diseases.
